# Binary Eluent Based Vortex-Assisted Matrix Solid-Phase Dispersion for the Extraction and Determination of Multicomponent from Musk by Gas Chromatography-Mass Spectrometry

**DOI:** 10.1155/2021/9913055

**Published:** 2021-08-12

**Authors:** Shanshan Wang, Ye Shang, Chunxiao Liang, Tao Liu, Kunze Du, Jiading Guo, Jin Li, Yan-xu Chang

**Affiliations:** ^1^State Key Laboratory of Component-Based Chinese Medicine, Tianjin University of Traditional Chinese Medicine, Tianjin 301617, China; ^2^Tianjin Key Laboratory of Phytochemistry and Pharmaceutical Analysis, Tianjin University of Traditional Chinese Medicine, Tianjin 301617, China

## Abstract

A green, flexible, and effective strategy was proposed to quantify four target compounds (muscone, ethyl palmitate, ethyl oleate, and ethylparaben) from musk by binary eluent based vortex-assisted matrix solid-phase dispersion (MSPD) extraction coupled with GC/MS. Single-factor tests and orthogonal design were employed to optimize the MSPD parameters. In addition, the binary eluent system, methanol, and ethyl acetate 3 : 7 (v/v) were used to extract the target analytes. Finally, *C*_18_ was applied as the easily available dispersant and the sample powder was ground for 2 min. Thereafter, the mixture was rapidly extracted with the binary eluents under whirling for 3 min. Eventually, the analysis of the samples was completed within 12 min by GC/MS. All correlation coefficients (*r*) of four targets were more than 0.9991. The recoveries of all target compounds ranged from 92.8% to 101% while their RSDs were less than 6.94%. There was no significant matrix interference for the analysis. Thus, the combination of vortex-assisted MSPD with GC/MS was considered as a novel, rapid, and environmentally friendly quantitative approach for musk samples.

## 1. Introduction

Musk, as a precious aromatic-type traditional medicine, is obtained from the dried secretion of mature male *Moschus berezovskii* Flerov*, Moschus sifanicus* Przewalski, and *Moschus moschiferus* Linnaeus principally distributed in Northwest and Northeast of China. It has a significant medicinal value that is mainly manifested in resuscitating and restoring consciousness. As a crucial monomer, muscone has made a great contribution to brain injury because it could enhance the permeability of the blood-brain barrier (BBB) [1] and protect the neurons [2] through prolonging the resistance to hypoxia. Moreover, it had good performance in the treatment of diabetic neuropathy, anti-inflammatory, and analgesic activities [3–5]. In addition, fatty acids (such as ethyl palmitate and ethyl oleate) could regulate blood lipid level, improve immunity, and resist oxidation [6–8]. In recent years, the shrinkage of suitable habitats made the numbers of wild musk dramatically reduce under the adverse effects of the natural environment and hunting [9]. The bad impact of the musk reduction led to the lack of natural musk. Therefore, it is of great necessity to enhance the management system of artificially culture musk and improve the quality of artificial musk. Currently, the study of quality assessment is more focused on muscone in musk and its Chinese patent medicines. It was less comprehensive and significant for the quality control of musk and its products. Moreover, the sample preparation of musk has faced huge challenges of low recovery, long time consuming, and low efficiency. Hence, it is of great significance to propose an effective approach to extract and determine the multicomponents in musk.

In order to gain convincing data, it is indispensable to perform a biosample processing method for multicomponent extraction and analysis. Traditional preparation methods such as ultrasonic vibration [10], heating reflux [11], and microwave [12] have been extensively used in traditional Chinese medicine (TCM) extraction. However, they are often followed by negative phenomenon such as sample waste, organic solvent and extraction time consumption, complex operation, and low efficiency. Although ultrasonic treatment [13] and soxhlet extraction [14] are often used preferentially for volatile or semivolatile components in TCM, the low recovery, organic solvents, and time-wasting still could not be avoided. Therefore, it was necessary to develop a rapid and environmentally friendly quantitative approach for musk samples.

The matrix solid-phase assist extraction (MSPD) method allowed the adequate contact of a few samples with minute quantities of fillers and the attendance of appropriate eluent. The extraction process is that the sample is ground by the dispersant or adsorbent to expose the target component [15]. The elution procedure is that the transferred analytes are eluted from dispersant or adsorbent. Therefore, MSPD presents outstanding performance on agricultural residues [16], cosmetics [17], and air pollution [18] with the highlights of green environmental protection, simple process, satisfactory recovery, and high efficiency. At the same time, it is rarely reported that MSPD is applied to volatile components in TCM [19]. Consequently, it is advisable to modify the traditional MSPD to make up for its disadvantages of extraction of volatile components from TCM.

Recently, MSPD was suitable for simple combination with other technologies such as the integration of ultrasound [20], vortex [21], and magnetic [22] to handle sample pretreatment problems. In particular, vortex is preferred as an auxiliary method with the characteristics of high-speed oscillation and high throughput. Typically, vortex-assisted MSPD has occupied a prominent position in the extraction of active ingredients including anthraquinone [23], iridoid [24], flavonoids [25], and phenolic acids [26] in TCMs. Therefore, it is considered that vortex, as an assisted tool, could be performed to rapidly acquire satisfactory recovery and extraction time. *C*_18_ filler possessed a strong retention ability for weak polar substances due to the long-chain effect [19]. Meanwhile, *C*_18_ provides considerable dispersion and adsorption of weakly polar components with the characteristics of low cost, easy access, green, nontoxic, and wide application. Furthermore, it is not enough to obtain all the target components with a single extraction solvent. However, it is critical to quantitatively multiple components for quality control of traditional Chinese medicine. The proposal of binary or multiple eluents effectively alleviates the situation. It is reflected that binary eluents give full play to their respective characteristics in order to increase the amount and contents of components and achieve the approving beneficial result. The presence of binary eluents provides the possibility of component diversity, which can increase the content of the target in single MSPD extraction.

Muscone was analyzed by high-performance liquid chromatography (HPLC) [27], ultra-performance liquid chromatography (UPLC) [28], and gas chromatography-mass spectrometry (GC/MS) [29] in musk. Among them, GC/MS is a separation and analysis technology characterized by its high sensitivity, high selectivity, high efficiency, and independent database. In particular, it has shown its unique advantages in the determination of volatile components. Nevertheless, it is persuasive that multicomponent quantification could better describe the quality, activity, and efficacy of traditional Chinese medicine.

This study aims to develop a novel, green, and efficient vortex-assisted MSPD method followed by GC/MS to extract and determine multiple compounds (muscone, ethyl palmitate, ethyl oleate, and ethylparaben) in musk. In addition, the binary solvents were employed to extract as many effective compounds as possible with different polarities from the packing. Moreover, the combination of vortex and MSPD simplifies the sample pretreatment and efficiently saves time. For the optimal extraction results, this study determined the specific parameters of vortex-assisted MSPD.

## 2. Material and Methods

### 2.1. Chemicals and Reagents

The organic reagents including methanol (HPLC-grade), ethanol (HPLC-grade), ethyl acetate (HPLC-grade), and n-hexane (HPLC-grade) were provided by Fisher (Leicestershire, UK) and Concord (Tianjin, China), and all of them were above 99% purity. Five standards (muscone, ethyl oleate, ethyl palmitate, ethylparaben, and isopsoralen) were provided by Deste (Chengdu, China) and the purity was higher than 99%. Isopsoralen (>99%) was selected as an internal standard (IS). Adsorbents including *C*_18_, *C*_8_, Florisil PR, HXN, and PEP were obtained from Welch Materials (Shanghai, China).

### 2.2. Preparation of Standard Solutions

**Figure 1:**
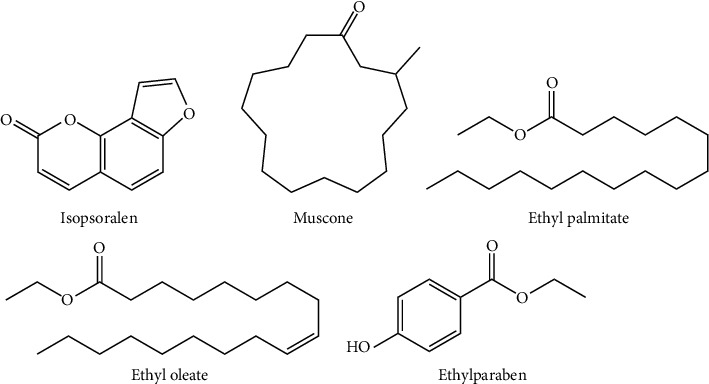
The structure of isopsoralen (IS) and multiple compounds in the samples.

The standard substances of muscone (boiling point 329.5°C at 760 mmHg and LogP 6.33), ethyl palmitate (boiling point 342.2°C at 760 mmHg and LogP 8.15), ethyl oleate (boiling point 385.9°C at 760 mmHg and LogP 8.69), ethylparaben (boiling point 297.5°C at 760 mmHg and LogP 2.40), and isopsoralen (boiling point 362.6°C at 760 mmHg and LogP 2.01, IS) (the chemical structures are shown in Figure 1) were accurately weighed and quantified with methanol at the concentrations of 2 mg·mL^−1^, 1 mg·mL^−1^, 2 mg·mL^−1^, 2 mg·mL^−1^, and 1 mg·mL^−1^, respectively. A series of mixed stock solutions were composed of muscone (0.4–100 *µ*g·mL^−1^), ethyl palmitate (0.08–20 *µ*g·mL^−1^), ethyl oleate (0.08–20 *µ*g·mL^−1^), and ethylparaben (0.16–40 *µ*g·mL^−1^). Then, it was used to prepare for calibration curve by stepwise dilution with methanol. Additionally, a certain quality of the internal standard substance (1 mg·mL^−1^) was added in the mixed working solutions and sample solutions. All stored solutions were placed at −20°C.

### 2.3. Chinese Patent Medicine

Two batches of artificially cultivated musk were collected from Qinghai and Tibet (China), and one batch of synthetic musk was purchased from Bozhou Medicinal Materials Market (Anhui, China). The remaining eight batches of Chinese patent medicines containing Musk were collected from two different pharmacies in Tianjin, including Xue-shuan-xin-mai-ning (XSXMN) capsule, Hong-hua-qi-li (HHQL) powder, Xiao-jin (XJ) pill, Xiao-er-qi-zhen (XEQZ) pill, She-xiang-bao-xin (SXBX) pill, and Wei-chang-an (WCA) pill, respectively. Eleven batches of samples were ground into a fine powder and passed through 50 meshes sieve for further research.

### 2.4. Binary Eluent Based Vortex-Assisted MSPD Microextraction Procedure

**Figure 2:**
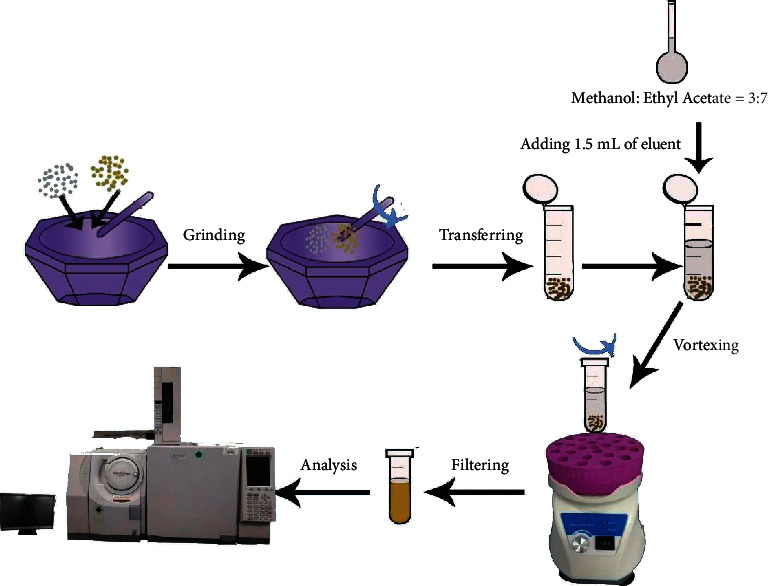
Schematic diagram of binary eluent based vortex-assisted MSPD.

As the schematic diagram (Figure 2) shows, XSXMN capsule powder (20 mg) and *C*_18_ dispersant (40 mg) were accurately weighed and gently put into the agate mortar. Immediately, the mixture was ground with the uniform force for 2 min, and then transferred as softly as possible into a 2 mL centrifuge tube. A binary extraction solvent was constituted of ethyl acetate and methanol at a 7 : 3 ratio. This homogeneous solvent was not prepared until used for extraction. The tube was shocked vigorously agitated by means of a vortex for 3 minutes. Finally, the entire extraction system was centrifuged at 140 g for 3 min, and the supernatant was passed through a 0.22 *μ*m miniature nylon membrane for further GC/MS analysis of the targets.

### 2.5. Soaking Extraction

In accordance with the authoritative 2020 edition Chinese Pharmacopoeia, musk was treated by soaking. A certain quantity of XSXMN capsule powder (20 mg) was accurately weighed and sealed carefully in a 2 mL centrifuge tube. Then, the mixture in 1 mL ethanol was thoroughly shaken and left standing for one hour. Subsequently, the extracted solutions were filtered through a 0.22 *μ*m nylon membrane. The final filtrate solution was ready for GC/MS analysis.

### 2.6. Ultrasonic-Assisted Extraction

Based on many studies, ultrasonic extraction for musk was considered to be the conventional method. The precisely weighed XSXMN capsule powder (20 mg) was put into a 2 mL centrifuge tube and seriously sealed. The mixture was sonicated for 30 min on ultrasonic equipment (300 W). Then, the extraction solution was cooled and replenished the weight loss. The mixed solution was passed through a nylon filter membrane (0.22 *μ*m) to obtain the clear solution for subsequent GC/MS analysis.

### 2.7. GC/MS Analysis

**Figure 3:**
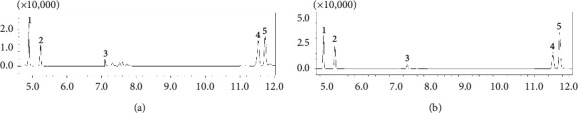
GC-MS under the SIM mode figure of extract of Xue-shuan-xin-mai-ning capsule on the vortex-assisted MSPD (a); mixed standard compounds (b). *Peaks*. 1: muscone; 2: ethyl palmitate; 3: ethyl oleate; 4: ethylparaben; 5: isopsoralen (IS).

A Shimadzu GC/MS-QP2010 Ultra (Shimadzu, Japan) gas chromatography-mass spectrometry was carried for the qualitative and quantitative analysis of the target analytes. An RTX-wax (0.32 mm, 0.32 mm, and 15 m) quartz capillary column was used for the chromatographic separation of the analytes. The device was operated at an inlet temperature of 250°C and an interface temperature of 230°C. Under split injection mode conditions, the carrier gas flow rate of high-purity helium gas was set to 2.8 mL/min for the separation of the target active ingredients. The setting conditions of 15 KPa of column pressure and split ratio (10 : 1) made the separation performance of the target analytes better. The temperature-programmed route was eventually optimized with the following procedure: initial oven temperature at 120°C, sloping at 8°C/min to 198°C and holding for 1 min, sloping at 1°C/min to 200°C, and finally sloping at 10°C/min to 240°C with 5 min of holding time. The parameters of electron impact ionization (EI) source were set as follows: ionization energy at 70 eV, temperature as 230°C, and the detector voltage as 1.5 kV. The mass range (m/z) was 40–400 with SCAN mode, and the scanning interval was 0.3 s. In SIM mode, quantitative m/z ratios of four compounds (muscone, ethyl palmitate, ethyl oleate, and ethylparaben) were 238/85, 238/125; 310/69, 310/97; 284/88, 284/101; and 166/65, 166/121, respectively. The target compounds and IS in all the samples were acquired by SCAN mode for identification and by SIM mode for quantitation, respectively. The separation of analytes and IS were obtained in the total ion chromatogram (TIC) under the above GC/MS Analysis conditions (Figure 3).

### 2.8. Optimization Experiment of Vortex-Assisted MSPD

Compared with the multivariable mathematical model, a single-factor experimental design could reflect the changing trend of a single factor. Single-factor experiments were first employed to define each specific parameter in the binary eluent based vortex-assisted MSPD. XSXMN capsule powder (20 mg·mL^−1^) was initially used as the object for the optimization of related conditions. Seven single-factor experiments were rigorously implemented and repeated three times. Dispersant type, ratio of sample to filler, grinding time, type of eluent, ratio of binary eluent, volume of binary eluent, and elution time were individually optimized, separately. The data of seven groups were analyzed independently by one-way ANOVA (SPSS statistics 17.0). It was shown with different letters representing significant differences. The relevant parameters of the MSPD program were initially established by the single-factor experiments, and the experimental results were subsequently described in detail.

Considering the total content of the analytes as a marker, the four-factor and three-level (*L*_9_3^4^) orthogonal design was proposed correspondingly for vortex-assisted MSPD. The four factors have participated in the orthogonal design, which were grinding time (*A*, min), eluent proportion (*B*), eluent volume (*C*, mL), and eluent time (*D*, min) based on XSXMN capsule powder (20 mg) and sample to *C*_18_ dispersant (1 : 2). The orthogonal header design and experimental scheme are shown in Table S1 (in Supplementary Material) and Table 1.

## 3. Results and Discussion

### 3.1. Single-Factor Experiment

#### 3.1.1. Type of Dispersant

**Figure 4:**
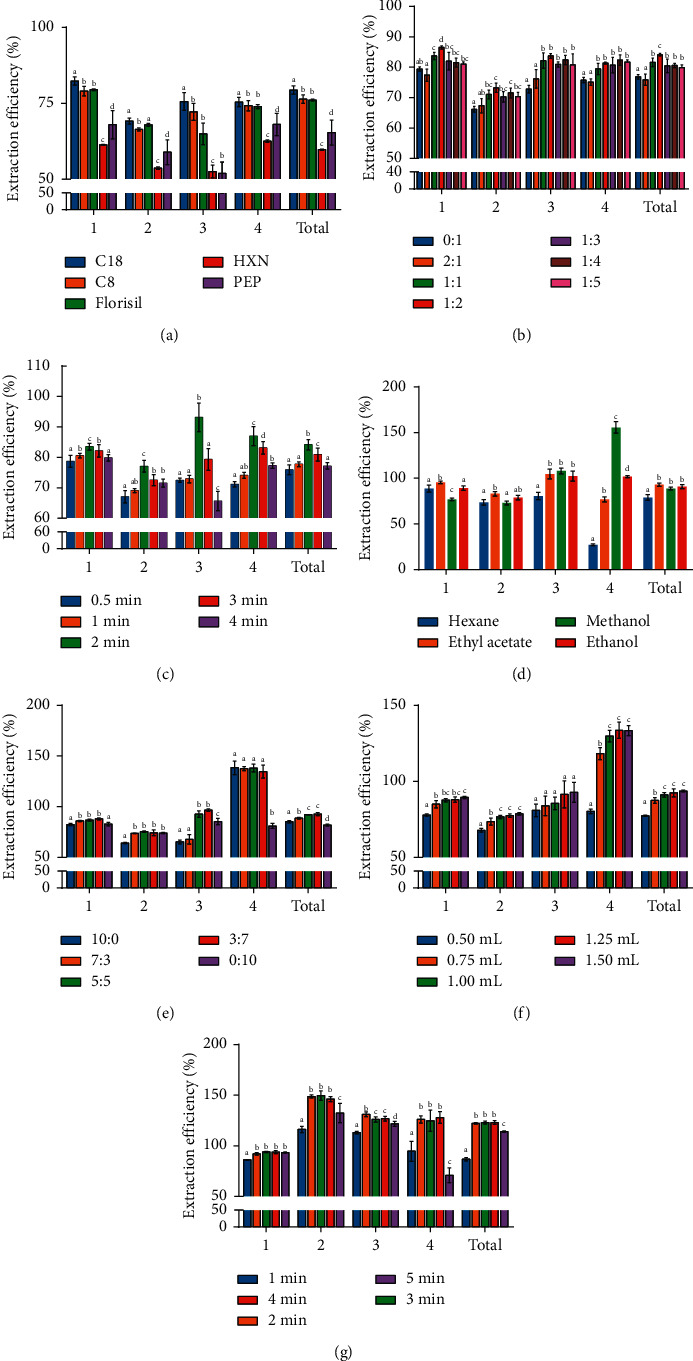
Effects of parameters on the efficiency of 4 contents. 1: muscone; 2: ethyl palmitate; 3: ethyl oleate; 4: ethylparaben. (a) Type of the dispersant. (b) Ratio of sample to dispersant. (c) Grinding time. (d) Type of eluent solvent. (e) Proportion of binary eluent. (f) Volume of binary eluent. (g) Vortex time.

In the MSPD step, the solid dispersant has two important effects on the sample including mechanical damage and adsorption capacity [19]. The analyte was exposed to the surface of the sample by grinding and dispersing the sample with dispersant, and the exposed substance was transferred by the dispersant. The interaction between the target compound and packing may be hydrogen bonding and van der Waals forces [27]. Therefore, *C*_18_, *C*_8_, Florisil, HXN, and PEP were investigated to select superior dispersants. Compared with the other four dispersants, *C*_18_ has resulted in a significant contribution (*P* < 0.05) to analyte extraction efficiency (Figure 4(a)). As a nonpolar stationary phase, *C*_18_ is octadecylsilane bonded silica gel packing with superior hydrophobic. All of the four compounds present the carbonyl structure, which were advantaged for interacting with *C*_18_ by hydrogen bonding. Therefore, it possible to exert retention effect on the four weak polar compounds. Moreover, *C*_18_ could further promote exposure and homogenization of the targets due to the strong mechanical properties. However, it was observed that the dispersion effect of the other four fillers was weaker than that of *C*_18_. *C*_8_ was a silica bonded octyl group, which is located in a weak polar position. Florisil may had a hydrogen bond with the target through the silicon hydroxyl, and the interaction force is slightly weak. As polymers, PEP and HXN showed poor response to the targets, and their loose texture resulted in significant errors. Finally, *C*_18_ was considered as the most suitable solid dispersant among the five adsorbents for the subsequent experiments due to its good dispersion and retention capacity.

#### 3.1.2. The Ratio of Sample to Dispersant

It was necessary for MSPD to make an in-depth study on the proportion of the dispersant and the sample powder in quantity due to the parameter influencing the extraction performance. The mass ratio of 1 : 0, 2 : 1, 1 : 1, 1 : 2, 1 : 3, 1 : 4, and 1 : 5 was then systematically examined (Figure 4(b)). The increased dispersant content resulted in increased exposure of the compounds within the mass ratio of 1 : 0 to 1 : 2, whereas the contents of the compounds started to show downward trends until 1 : 3. It is presumably surmised that the higher ratio of *C*_18_ could increase the contact area with the powder, and the targets were easily transferred to the filler from the powder under strong mechanical force. A high proportion of filler is not capable of acquiring better results, yet being counterproductive. The extraction efficiency of the target substance was obstructed by excessive fillers owing to its own retention and dispersion ability. Combining the results, the ratio of the amount of the dispersant to the sample was specified as 1 : 2.

#### 3.1.3. Grinding Time

The grinding time was equally focused on the content of the target in the vortex-assisted MSPD program. To obtain satisfactory results of analyte content, a series of grinding times of 0.5 min, 1 min, 2 min, 3 min, and 4 min were observed in the study (Figure 4(c)). The tendency is that the extraction efficiency is initially increased and then decreased on the whole. The increased route (0.5–2 min) was possible to explain that the powder and filler molecules fully collided through the agate mortar rod, and the grinding causes the target compounds to be better released from the sample; the decreased route (2–4 min) is that the physical structure of *C*_18_ was destroyed, so the adsorption capacity of the filler was reduced. Comprehensively, grinding for 2 min was recognized to be propitious to extract four compounds in the study.

#### 3.1.4. Type of Eluent Solvent

Another key parameter, different types of eluents, was characterized by the diverse ability of affinity to analytes. Therefore, the experiment list was assigned four solvents including methanol, ethanol, ethyl acetate, and n-hexane to get the desired effect (Figure 4(d)). Compared with other eluents, the maximum content of ethylparaben was obtained with elution of methanol, whereas the other three substances were eluted with ethyl acetate to the maximum extent. Ethylparaben, as a polar compound, was easily eluted by methanol that was similar to methanol in polar property to get a prominent phenomenon. Similarly, as weak polar substances, muscone, ethyl palmitate, and ethyl oleate were more suitable to be eluted by a weak polar eluent. The other two elutes n-hexane and ethanol, provided poor elution results. Taking great polarity difference of analytes into account, methanol and ethyl acetate were together selected as binary eluent for further optimization.

#### 3.1.5. Proportion of Binary Eluent

The volume proportion between methanol and ethyl acetate was directly related to the ability of the binary eluent. It was nonnegligible to explore the volume ratio at five levels containing 10 : 0, 7 : 3, 5 : 5, 3 : 7, and 0 : 10. The results (Figure 4(e)) showed that the amount of ethylparaben eluted without methanol decreased dramatically. It may be caused that the weak polarity of ethyl acetate was not enough to adequately elute ethylparaben. Moreover, the contents of all components showed a downward trend with the ratio of ethyl acetate until 0 : 10, which were caused by the high volatile of ethyl acetate. At the level of 3 : 7, not only the extraction efficiency of each component but also the total content reached the maximum. It was concluded that the proportion of methanol and ethyl acetate at 3 : 7 was finally selected to serve for the binary eluents in the project.

#### 3.1.6. Volume of Binary Eluent

It is equally momentous to appraise the volume of binary eluent in a vortex-assisted MSPD program. The target compounds were extracted from the surface of the filler by an adequate volume of the eluent. The sequences of 0.5 mL, 0.75 mL, 1 mL, 1.25 mL, and 1.5 mL were studied to obtain better results. The yield of each target compound increased until the volume of binary eluent was up to 1.25 mL (Figure 4(f)). The phenomenon was explained that the binary eluent volume was at a certain value and the target substance was desorbed cleanly from the filler. It was found that there is no significant difference between 1.25 mL and 1.5 mL. Hence, the binary eluent volume was determined as 1.25 mL, satisfying the extraction of all target compounds.

#### 3.1.7. Vortex Time

As an auxiliary means, the vortex extraction could force the targets to be rapidly exposed from the packing. Vortex time was investigated at different levels (1 min, 2 min, 3 min, 4 min, and 5 min) in the project (as shown in Figure 4(g)). The ANOVA analysis revealed no significant difference in total content within the range of 2–4 min, suggesting that the targets were sufficiently extracted during the period. The decrease of total content appeared at 5 min followed by the loss of target compounds due to eluent volatility. Ultimately, 2 min was considered as the optimal vortex time to force the MSPD procedure.

### 3.2. Orthogonal Design

As a multifactor experimental design, the orthogonal design results are shown in Table S1, including grinding time (1 min, 2 min, and 3 min), binary eluent proportion (methanol: ethyl acetate = 5 : 5, 3 : 7, 1 : 9), eluent volume (1.00 mL, 1.25 mL, and 1.50 mL), and vortex time (1 min, 2 min, and 3 min). With the help of the orthogonal design, the total content is analyzed intuitively. The *K* value represents the average value of each level of each factor, and the *R* value means the difference between the maximum value and the minimum value of each level. The magnitude of the *R* value determines the rank of the four factors in this work. Therefore, the ranking of the four factors was *B* > *A* > *D* > *C*. According to the principle of the higher *K*_*i*_ value, the experimental scheme was initially identified as *A*_2_*B*_2_*C*_3_*D*_3_ (Table 1). Furthermore, the experimental results were statistically analyzed on account of the optimal scheme by SPSS software (Table S2 in Supplementary Material). Both A and B factors were performed statistically significantly (*P* < 0.05), and the levels were defined as *A*_2_*B*_2_. As a result of *P* > 0.05, three levels of the other two factors were considered to be undifferentiated, and the levels were considered preliminary as *A*_2_*B*_2_C_0_*D*_0_. To maximize the benefits of total content without wasting time and solvents, the combination of *A*_2_*B*_2_C_3_*D*_3_ (grinding for 2 min, methanol and ethyl acetate 3 : 7 (v/v), elution volume 1.5 mL, vortex for 3 min) was determined as the final experimental scheme, corresponding to the results of a single factor.

### 3.3. Method Validation

To verify the feasibility of this work, a series of method validation was implemented in the vortex-assisted MSPD, including the standard curve, LODs, LOQs, matrix effect, and the recovery rate (Table 2). The standard curves for muscone (0.4–100 *μ*g·mg^−1^), ethyl palmitate (0.08–20 *μ*g·mL^−1^), ethyl oleate (0.08–20 *μ*g·mL^−1^), and ethylparaben (0.16–40 *μ*g·mL^−1^) were plotted with eight concentrations of mixed reference substances and 1 *μ*g·mL^−1^ of IS was added, which was used to obtain the standard equation of four compounds. The correlation coefficients of the four standard curves were all above 0.9991 and tended to 1, which explained a satisfying linear relationship. The theoretical value was in better agreement with the experimental value. The limit of detection (LOD) and limit of quantification (LOQ) were the corresponding target concentrations at a signal-to-noise ratio of three and ten times, respectively. The order of magnitude of LOD and LOQ was expressed as the nanogram level, and their maximum values are only 16.8 ng·mL^−1^ and 56.2 ng·mL^−1^, respectively. It is necessary to evaluate the matrix effect of four targets in this work. The matrix effect is to evaluate the degree of influence of the matrix on the analysis process of the analyte. It is calculated to compare the peak area of the spiked extraction solution (*A*) and the spiked matrix (*B*) [30]. The evaluation formula of the matrix effect was as follows:

(1) $$\begin{array}{c} \mathrm{Matrix effect}\;\left(\%\right)=\frac{B-A}{A}\times 100. \end{array}$$

It was generally considered that the matrix had no significant interference with the analysis of analytes and the analysis results have certain accuracy within 20%. The enrichment factor (EF) was calculated by comparing the contents of the four target components after the vortex-assisted MSPD program with the contents of untreated standard solutions [31]. The recovery was the actual amount of analyte that was used to analyze the sample. In other words, it was used to assess the amount of analyte lost through sample processing. The recoveries of the targets were 92.8%–101%, and the RSD% was relatively acceptable, ranging from 3.11 to 6.94%. It may be related to the volatile characteristics of the solvents and analytes.

The reliability of the method was further verified by three projects including interday precision, intraday precision, and accuracy at low, medium, and high levels (Table S3 in Supplementary Material). At three levels, the interday precision (RSD% < 5.7%) and intraday precision (RSD% < 5.9%) of the four compounds were adapted to the analysis standard, respectively. The analytes were tested for 24 h of stability, and RSD% was below 6.1%. To sum up, it resulted in a simple, stable method.

### 3.4. Application

The eight batches of Chinese patent medicine and three batches of artificial musk containing four target compounds were carried out by optimizing the optimal conditions in the vortex-assisted MSPD procedure. The analysis results of the collected actual samples are arranged (Table 3). Chinese patent medicine was a compound preparation, which was composed of one or more Chinese herbal medicines. Four compounds (muscone, ethyl palmitate, and ethyl oleate, and ethylparaben) were detected in eight batches of Chinese patent medicines containing musk. The mass of musk in an individual sample varied, and muscone was the main ingredient for musk with a content of 0.023–2.259 *μ*g·mg^−1^ in the samples. Moreover, the range from 0.035 to 73.397 *μ*g·mg^−1^ was quantified in artificial musk and cultured *Moschus berezovskii*. The difference in muscone content between the two groups of *Moschus berezovskii* was exceedingly notable, which is mainly due to the time of collecting musk. The lower side was collected as the secreted musk for less than three months, and the higher side was collected for one year. Ethylparaben was not detected in musk which is less than three months. The contents of the other three components are inconsistent in several batches of samples, ranging from 0.010 to 0.854 *μ*g·mg^−1^ (ethyl palmitate), 0.031–2.410 *μ*g·mg^−1^ (ethyl oleate), and 0.009–20.279 *μ*g·mg^−1^ (ethylparaben), separately. It was reported that musk was extracted by ultrasonic treatment with high efficiency in recent years. The standard extraction method of the 2020 edition of Chinese pharmacopoeia was to leave for one hour for musk. To highlight the advantages of this work, the above two extraction methods were implemented for statistical analysis (*t*-test) with vortex-assisted MSPD. Taking XSXMN capsule as an example, vortex-assisted MSPD has the primary preponderance with a significant difference (*P* < 0.05), compared with the other two strategies. The order of their extraction effect was vortex-assisted MSPD > ultrasound extraction > static extraction. All in all, the proposed MSPD is comprehensively evaluated as the advanced extraction method of environmental protection because of the advantages of a small amount of organic solvent and sample and short extraction time.

### 3.5. Assessment of Method Greenness

**Figure 5:**
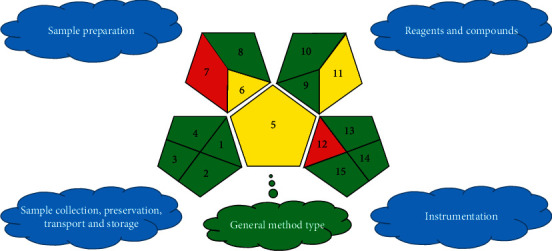
GAPI assessment of the green profile of the evaluated procedures for determining four targets in musk and its Chinese patents.

It is inevitable for the environment and people to pose threats such as hazardous reagents, waste liquids, energy, and pollution in the research process. Hence, it is very necessary for analytical methods to evaluate the green index in the green analytical chemistry field. Therefore, an analytical ecoscale was introduced by Gałuszka et al. [32]. The evaluation procedure is calculated on a 100-point basis through the penalty system. The study ought to be comparatively green with a total score of 89 points (Table S4). Nevertheless, sample preparation and hazard generation were absent for ecoscale evaluation methods. Recently, the green analytical procedure index (GAPI) was proposed by Płotka-Wasylka [33]. The evaluation of the green index starts from three aspects: sample preparation, reagents and solvents, and instruments, and they are subdivided into 15 dots to visually show the greenness of the method in pictograms. The visual pictogram is capable of observing directly the greenness for current research (Figure 5). The result is consistent with the ecoscale evaluation method. Consequently, it is highly dependable to evaluate the method by two different green indexing systems.

### 3.6. Comparison with Other Reported Methods

A specific comparison of this proposed method was shown (Table S5) with other published related methods [27–29, 34]. It was an excellent performance of vortex-assisted MSPD that presented a short extraction time and fewer organic solvents for rare TCM. It effectively avoided the shortcoming of low efficiency and the great demand for samples and organic solvents in the ultrasonic and soaping extraction strategies. On the contrary, vortex-assisted MSPD has achieved high efficiency, accuracy, and green contribution in the whole process of extraction and detection. Consequently, the new vortex-assisted MSPD was a novel, rapid, and environmentally friendly quantitative approach for musk samples.

## 4. Conclusions

A binary eluent based vortex-assisted MSPD combining with GC/MS strategy has been successfully applied to extract and determine the multiple compounds in a variety of medicine including Chinese patent medicine, breeding musk, and artificial musk. The dispersant *C*_18_ packing (the characteristics of reducing interference) was implemented to extract muscone, ethyl palmitate, ethyl oleate, and ethylparaben. The binary coordination of methanol and ethyl acetate made an important contribution to the improvement of the amount of four target substances with the performance of low use of organic solvents. The auxiliary of vortex greatly shortens the extraction time and avoids the volatilization of compounds. Moreover, the great accuracy, precision, and rationality of the method are obtained by a rigorous investigation. In general, *C*_18_ packing and sample powder (1 : 2), grinding for 2 min, 1.5 mL of binary eluent (methanol: ethyl acetate = 3 : 7), and vortex for 3 min were selected as the reference for the sample pretreatment process, and further quantitative analysis was carried out by GC/MS. The strategy was a potential extraction method in volatile substances, which has the merits of environmental friendliness, short time, low toxicity, and high extraction efficiency. In addition, it was necessary to consider the recycle of the dispersants further to improve the sustainability of the dispersant material in the subsequent MSPD procedure. The proposed vortex-assisted MSPD with GC/MS was considered as a novel, rapid, and environmentally friendly quantitative approach for musk samples.

## Figures and Tables

**Table 1:** Optimization of the results of the orthogonal experimental design.

No.	Grinding time (*A*) (min)	Eluent ratio (*B*) (methanol: ethyl acetate)	Elution volume (*C*) (mL)	Elution time (*D*) (min)	Total content (mg·g^−1^)
1	1	1	1	1	0.36 ± 0.02
2	1	2	2	2	0.36 ± 0.01
3	1	3	3	3	0.33 ± 0.01
4	2	1	2	3	0.39 ± 0.01
5	2	2	3	1	0.38 ± 0.01
6	2	3	1	2	0.34 ± 0.01
7	3	1	3	2	0.37 ± 0.01
8	3	2	1	3	0.37 ± 0.01
9	3	3	2	1	0.33 ± 0.01
*K*1	0.35	0.37	0.36	0.35	
*K*2	0.37	0.37	0.36	0.36	
*K*3	0.36	0.33	0.36	0.36	
*R*	0.02	0.04	0.01	0.01	
Order	*B* > *A* > *D* > *C*				
Optimal level	*A*_2_	*B*_2_	*C*_3_	*D*_3_	
Optimal combination	*A*_2_ *B*_2_ *C*_3_ *D*_3_				

*K*_*i*_: the average of each level in each factor. *R*: the difference between the maximum and minimum levels of each factor.

**Table 2:** The related results of method validation for the target compounds by the vortex assisted MSPD.

Compounds	Regression equation	*r*	Linearity range (*µ*g·mL^−1^)	LOD (ng·mL^−1^)	LOQ (ng·mL^−1^)	Matrix effect (%)	Enrichment factors	Recovery (*n* = 6)	
								Average (%)	RSD (%)
Muscone	*y* = 0.53*x* − 0.05	0.9998	0.4–100	4.44	14.8	−17	1.1	96.5	3.11
Ethyl palmitate	*y* = 1.86*x* − 0.09	0.9993	0.08–20	1.76	5.76	−16	1.2	94.2	6.94
Ethyl oleate	*y* = 0.40*x* − 0.04	0.9991	0.08–20	12.7	42.4	−13	1.5	92.8	3.81
Ethylparaben	*y* = 2.26*x* − 0.05	0.9997	0.16–40	16.8	56.2	−15	1.4	101	4.17

**Table 3:** The contents of four compounds in 8 batches of Chinese patent medicine and 3 batches of musk samples (mean ± SD, *n* = 3).

Batches	Muscone (*μ*g·mg^−1^)	Ethyl palmitate (*μ*g·mg^−1^)	Ethyl oleate (*μ*g·mg^−1^)	Ethylparaben (*μ*g·mg^−1^)
XSXMN capsule^a^	0.236 ± 0.003	0.041 ± 0.001	0.047 ± 0.001	0.049 ± 0.002
XSXMN capsule^b^	0.201 ± 0.005	0.033 ± 0.001	0.031 ± 0.001	0.035 ± 0.002
XSXMN capsule^c^	0.209 ± 0.003	0.035 ± 0.001	0.032 ± 0.001	0.036 ± 0.001
XJ pill	0.023 ± 0.001	0.074 ± 0.001	0.184 ± 0.006	0.042 ± 0.001
HHQL powder	2.259 ± 0.110	0.024 ± 0.001	0.207 ± 0.009	0.452 ± 0.011
XEQZ pill	0.142 ± 0.001	0.018 ± 0.001	0.066 ± 0.002	0.009 ± 0.001
SXBX pill	1.615 ± 0.065	0.846 ± 0.024	0.457 ± 0.013	0.424 ± 0.012
SXBX pill	1.538 ± 0.034	0.854 ± 0.018	0.472 ± 0.010	0.420 ± 0.007
WCA pill	0.295 ± 0.010	0.010 ± 0.001	0.077 ± 0.002	0.038 ± 0.001
WCA pill	0.290 ± 0.004	0.010 ± 0.001	0.070 ± 0.003	0.035 ± 0.001
Artificial cultivated musk (Tibet)	0.035 ± 0.001	0.027 ± 0.001	0.529 ± 0.022	ND
Artificial cultivated musk (Qing hai)	18.572 ± 0.434	0.092 ± 0.003	2.410 ± 0.074	0.014 ± 0.001
Artificial musk	73.397 ± 1.231	0.377 ± 0.007	0.717 ± 0.012	20.279 ± 0.328

Note: Xue-shuan-xin-mai-ning (XSXMN) capsule, Hong-hua-qi-li (HHQL) powder, Xiao-jin (XJ) pill, Xiao-er-qi-zhen (XEQZ) pill, She-xiang-bao-xin (SXBX) pill, and Wei-chang-an (WCA) pill. ^a^XSXMN capsule was extracted by vortex assisted-MSPD. ^b^XSXMN capsule was extracted by soaking. ^c^XSXMN capsule was extracted ultrasonically.

## Data Availability

All the datasets presented in this study are included in the article.

## Supplementary Materials

Supplementary Materials *Table S1*. The factors and levels of the orthogonal experimental design. *Table S2*. The results of analysis of variance in the orthogonal experiment. *Table S3*. Intraday and interday precision and stability of target compounds. *Table S4*. The penalty points (PPs) for four-compound determination by vortex MSPD in musk and its Chinese patent medicine. *Table S5*. Comparison of the vortex-synchronized MSPD method with other methods in the determination of compounds in the musk.

## References

[1] Wang G.-Y., Wang N., Liao H.-N. (2015). Effects of muscone on the expression of P-gp, MMP-9 on blood-brain barrier model in vitro. *Cellular and Molecular Neurobiology*.

[2] Si Y.-C., Li Q., Xie C.-E., Niu X., Xia X.-H., Yu C.-Y. (2014). Chinese herbs and their active ingredients for activating xue (blood) promote the proliferation and differentiation of neural stem cells and mesenchymal stem cells. *Chinese Medicine*.

[3] Dong J., Li H., Bai Y., Wu C. (2019). Muscone ameliorates diabetic peripheral neuropathy through activating AKT/mTOR signalling pathway. *Journal of Pharmacy and Pharmacology*.

[4] Du Y., Gu X., Meng H (2018). Muscone improves cardiac function in mice after myocardial infarction by alleviating cardiac macrophage-mediated chronic inflammation through inhibition of NF-*κ*B and NLRP3 inflammasome. *American Journal of Tourism Research*.

[5] Zhao L., Hu C., Zhang P., Jiang H., Chen J. (2018). Novel preconditioning strategies for enhancing the migratory ability of mesenchymal stem cells in acute kidney injury. *Stem Cell Research & Therapy*.

[6] Yan M., Cai W. B., Hua T. (2020). Lipidomics reveals the dynamics of lipid profile altered by omega‐3 polyunsaturated fatty acid supplementation in healthy people. *Clinical and Experimental Pharmacology and Physiology*.

[7] Meijer K., De Vos P., Priebe M. G. (2010). Butyrate and other short-chain fatty acids as modulators of immunity: what relevance for health?. *Current Opinion in Clinical Nutrition and Metabolic Care*.

[8] Filannino P., Tlais A. Z. A., Morozova K. (2020). Lactic acid fermentation enriches the profile of biogenic fatty acid derivatives of avocado fruit (Persea americana Mill.). *Food Chemistry*.

[9] Jiang F., Zhang J., Gao H. (2020). Musk deer (Moschus spp.) face redistribution to higher elevations and latitudes under climate change in China. *The Science of the Total Environment*.

[10] Sicaire A.-G., Vian M. A., Fine F., Carré P., Tostain S., Chemat F. (2016). Ultrasound induced green solvent extraction of oil from oleaginous seeds. *Ultrasonics Sonochemistry*.

[11] Guo H., Liu A.-H., Li L., Guo D.-A. (2007). Simultaneous determination of 12 major constituents in Forsythia suspensa by high performance liquid chromatography-DAD method. *Journal of Pharmaceutical and Biomedical Analysis*.

[12] Li D., Sun C., Yang J. (2019). Ionic liquid-microwave-based extraction of biflavonoids from selaginella sinensis. *Molecules*.

[13] Lin X., Yi X., Ni S. (2021). Optimization of ultrasonic-assisted extraction and fatty acid composition of oil from paeonia suffruticosa andr. Seed. *Journal of Oleo Science*.

[14] Ayalew A. A. (2020). Chromatographic and spectroscopic determination of solvent-extracted Lantana camara leaf oil. *Journal of International Medical Research*.

[15] García-López M., Canosa P., Rodríguez I. (2008). Trends and recent applications of matrix solid-phase dispersion. *Analytical and Bioanalytical Chemistry*.

[16] Kemmerich M., Demarco M., Bernardi G., Prestes O. D., Adaime M. B., Zanella R. (2020). Balls-in-tube matrix solid phase dispersion (BiT-MSPD): an innovative and simplified technique for multiresidue determination of pesticides in fruit samples. *Journal of Chromatography A*.

[17] Chen M., Bai H., Zhai J. (2019). Comprehensive screening of 63 coloring agents in cosmetics using matrix solid-phase dispersion and ultra-high-performance liquid chromatography coupled with quadrupole-Orbitrap high-resolution mass spectrometry. *Journal of Chromatography A*.

[18] Śmiełowska M., Zabiegała B. (2019). Matrix solid-phase dispersion (MSPD) as simple and useful sample preparation technique for determination of polybrominated diphenyl ethers (PBDEs) in dust. *Analytica Chimica Acta*.

[19] Wianowska D., Dawidowicz A. L. (2016). Can matrix solid phase dispersion (MSPD) be more simplified? Application of solventless MSPD sample preparation method for GC-MS and GC-FID analysis of plant essential oil components. *Talanta*.

[20] Barfi B., Asghari A., Rajabi M., Barfi A., Saeidi I. (2013). Simplified miniaturized ultrasound-assisted matrix solid phase dispersion extraction and high performance liquid chromatographic determination of seven flavonoids in citrus fruit juice and human fluid samples: hesperetin and naringenin as biomarkers. *Journal of Chromatography A*.

[21] Chung W.-H., Lin J.-S., Ding W.-H. (2019). Dual-vortex-assisted matrix solid-phase dispersion coupled with isotope-dilution ultrahigh-performance liquid chromatography-high resolution mass spectrometry for the rapid determination of parabens in indoor dust samples. *Journal of Chromatography A*.

[22] Zhang H., Lai H., Wu X., Li G., Hu Y. (2020). CoFe_2_O_4_@HNTs/AuNPs substrate for rapid magnetic solid-phase extraction and efficient SERS detection of complex samples all-in-one. *Analytical Chemistry*.

[23] Jiang L., Wang J., Zhang H., Liu C., Tang Y., Chu C. (2019). New vortex-synchronized matrix solid-phase dispersion method for simultaneous determination of four anthraquinones in cassiae semen. *Molecules*.

[24] Du K., Li J., Bai Y., An M., Gao X.-M., Chang Y.-X. (2018). A green ionic liquid-based vortex-forced MSPD method for the simultaneous determination of 5-HMF and iridoid glycosides from Fructus Corni by ultra-high performance liquid chromatography. *Food Chemistry*.

[25] Xu J.-J., Yang R., Ye L.-H. (2016). Application of ionic liquids for elution of bioactive flavonoid glycosides from lime fruit by miniaturized matrix solid-phase dispersion. *Food Chemistry*.

[26] Chen S.-J., Du K.-Z., Li J., Chang Y.-X. (2020). A chitosan solution-based vortex-forced matrix solid phase dispersion method for the extraction and determination of four bioactive constituents from Ligustri Lucidi Fructus by high performance liquid chromatography. *Journal of Chromatography A*.

[27] Huang M., Xu W., Zhang Y. (2016). Identification and quantification of the anti-inflammatory constituents in Pian-Tze-Huang by liquid chromatography combined with quadrupole time-of-flight and triple quadrupole mass spectrometry. *Journal of Chromatography B*.

[28] Xu W., Zhang Y., Zhou C. (2017). Simultaneous quantification six active compounds in rat plasma by UPLC-MS/MS and its application to a pharmacokinetic study of Pien-Tze-Huang. *Journal of Chromatography B*.

[29] Chang W., Han L., Huang H. (2014). Simultaneous determination of four volatile compounds in rat plasma after oral administration of Shexiang Baoxin Pill (SBP) by HS-SPDE-GC-MS/MS and its application to pharmacokinetic studies. *Journal of Chromatography B*.

[30] Pano-Farias N. S., Ceballos-Magaña S. G., Muñiz-Valencia R., Jurado J. M., Alcázar Á., Aguayo-Villarreal I. A. (2017). Direct immersion single drop micro-extraction method for multi-class pesticides analysis in mango using GC-MS. *Food Chemistry*.

[31] Chatzimitakos T., Samanidou V., Stalikas C. D. (2017). Graphene-functionalized melamine sponges for microextraction of sulfonamides from food and environmental samples. *Journal of Chromatography A*.

[32] Gałuszka A., Migaszewski Z. M., Konieczka P., Namieśnik J. (2012). Analytical eco-scale for assessing the greenness of analytical procedures. *TRAC Trends in Analytical Chemistry*.

[33] Płotka-Wasylka J. (2018). A new tool for the evaluation of the analytical procedure: green analytical procedure index. *Talanta*.

[34] Tang Z.-S., Liu Y.-R., Lv Y. (2018). Quality markers of animal medicinal materials: correlative analysis of musk reveals distinct metabolic changes induced by multiple factors. *Phytomedicine*.

