# Supplementary material for: Binary Eluent Based Vortex-Assisted Matrix Solid-Phase Dispersion for the Extraction and Determination of Multicomponent from Musk by Gas Chromatography-Mass Spectrometry
Source: J Anal Methods Chem. 2021 Aug 12;2021:9913055. doi: 10.1155/2021/9913055 (PMC8378966; doi:10.1155/2021/9913055)
Supplement: Supplementary Materials — Table S1. The factors and levels of the orthogonal experimental design. Table S2. The results of analysis of variance in the orthogonal experiment. Table S3. Intraday and interday precision and stability of target compounds. Table S4. The penalty points (PPs) for four-compound determination by vortex MSPD in musk and its Chinese patent medicine. Table S5. Comparison of the vortex-synchronized MSPD method with other methods in the determination of compounds in the musk. [file 9913055.f1.docx]

**Supplementary Material**

**Binary eluent based-vortex assisted-matrix solid-phase dispersion for the extraction and determination of multi-component from musk by gas chromatography-mass spectrometry**

**Shanshan Wang^1, 2^, Ye Shang^1^, Tao Liu^1^, Kunze Du^1^, Jiading Guo^1^, Jun He^1^_,_ Jin Li^1*^, and Yan-xu Chang^1, 2*^**

^1^ State Key Laboratory of Component-based Chinese Medicine, Tianjin University of Traditional Chinese Medicine, Tianjin, 301617, China

^2^ Tianjin Key Laboratory of Phytochemistry and Pharmaceutical Analysis, Tianjin University of Traditional Chinese Medicine, Tianjin, 301617, China

*Corresponding author:

Yan-xu Chang*, State Key Laboratory of Component-based Chinese Medicine, Tianjin Key Laboratory of Phytochemistry and Pharmaceutical Analysis, Tianjin University of Traditional Chinese Medicine, Tianjin, 301617, China

**Tel.:** +86-022-59596163

**Fax:** +86-022-59596163

**E-mail**: Lijin@tjutcm.edu.cn (J. Li);Tcmcyx@tjutcm.edu.cn (Y.-x. Chang)

**Table S1.** The factors and levels of the orthogonal experimental design

| Factors | Grinding time(A)  (min) | Eluent ratio (B)  (methanol: ethyl acetate) | Elution volume (C)  (mL) | Elution time (D)  (min) |
| --- | --- | --- | --- | --- |
| Levels | 1 | 9:1 | 1.00 | 1 |
|  | 2 | 7:3 | 1.25 | 2 |
|  | 3 | 5:5 | 1.50 | 3 |

**Table S2.** The results of analysis of variance in the orthogonal experiment

| Factors | SS | df | MS | F | P value |
| --- | --- | --- | --- | --- | --- |
| A | 0.00 | 2 | 0.00 | 12.59 | 3.80×10^-4^* |
| B | 0.01 | 2 | 0.01 | 75.00 | 1.86×10^-9^* |
| C | 7.86×10^-5^ | 2 | 3.93×10^-5^ | 0.54 | 0.59 |
| D | 3.71×10^-4^ | 2 | 1.85×10^-4^ | 2.56 | 0.11 |
| Error | 0.00 | 18 | 7.25×10^-5^ |  |  |

* Significant difference (P<0.05)

**Table S3.** Intra-day and Inter-day precision, stability of target compounds

| Compounds | Concentration (µg mL^-1^) | Intra-day (n=6) | | Inter-day (n=6) | | Stability for 24 h | |
| --- | --- | --- | --- | --- | --- | --- | --- |
|  |  | Accuracy (%) | RSD (%) | Accuracy (%) | RSD (%) | Remains (%) | RSD (%) |
| Muscone | 0.8 | 96.5 | 4.20 | 95.1 | 3.73 | 96.2 | 4.85 |
|  | 10 | 102 | 3.50 | 100.5 | 2.99 | 99.5 | 3.32 |
|  | 50 | 103 | 3.03 | 102.3 | 3.12 | 102.2 | 3.30 |
| Ethyl palmitate | 0.16 | 101 | 5.30 | 98.1 | 5.34 | 98.0 | 4.36 |
|  | 2 | 96.5 | 3.72 | 96.4 | 3.77 | 96.7 | 3.95 |
|  | 10 | 98.3 | 4.63 | 98.8 | 3.15 | 99.3 | 3.26 |
| Ethyl oleate | 0.16 | 99.1 | 5.69 | 99.4 | 5.87 | 99.5 | 2.92 |
|  | 2 | 99.9 | 3.97 | 100 | 3.94 | 100 | 3.50 |
|  | 10 | 103 | 3.57 | 104 | 4.32 | 104.1 | 5.68 |
| Ethylparaben | 0.32 | 97.6 | 5.52 | 98.3 | 5.19 | 98.5 | 6.06 |
|  | 4 | 97.0 | 3.74 | 97.0 | 3.28 | 97.4 | 3.77 |
|  | 20 | 98.1 | 3.84 | 98.3 | 4.47 | 98.0 | 3.91 |

**Table S4.** The penalty points (PPs) for four compounds determination by vortex-MSPD in musk and its Chinese patent medicine

| Reagents | Penalty points |
| --- | --- |
| methanol | 1 |
| ethyl acetate | 2 |
| C_18_ | 0 |
|  | Σ3 |
| Instruments |  |
|  | Penalty points |
| GC-MS | 2 |
| vortex mixer | 0 |
| Waste | 3 |
| Occupational hazard | 3 |
|  | Σ8 |
| Total penalty points:11 |  |
| Analytical Eco-Scale total score: 89 |  |

**Table S5.** Comparison of the vortex- synchronized MSPD method with other methods in the determination of compounds in the musk

| No. | Extracted compounds | Samples | Samples amounts (mg) | Type of solvent | Solvent volume (mL) | Extraction method | Extraction time (min) | Detection method | Reference |
| --- | --- | --- | --- | --- | --- | --- | --- | --- | --- |
| 1 | muscone | Pien-TzeHuang | 120 | methanol | 100 | ultrasonic | 30 | HPLC–MS/MS | [27] |
| 2 | muscone | Pien-TzeHuang | 50 | methanol | 50 | ultrasonic | 30 | UPLC–MS/MS | [28] |
| 3 | muscone | She-xiang Bao-xin Pill | 200 | ethyl acetate | 2 | ultrasonic | 30 | HS-SPDE-GC  –MS/MS | [29] |
| 4 | muscone | Musk | 200 | ethanol | 2 | soaking | 60 | GC-MS | [34] |
| 5 | Muscone  ethyl palmitate  ethyl oleate  ethylparaben | Musk and other Chinese patent medicines | 20 | methanol and ethyl acetate (3:7) | 1.5 | vortex assisted MSPD | 3 | GC-MS | This study |
